# Lessons Learnt From the Experiences of Primary Care Physicians Facing COVID-19 in Benin: A Mixed-Methods Study

**DOI:** 10.3389/frhs.2022.843058

**Published:** 2022-03-29

**Authors:** Kéfilath Bello, Jan De Lepeleire, Christian Agossou, Ludwig Apers, Djimon Marcel Zannou, Bart Criel

**Affiliations:** ^1^Centre de Recherche en Reproduction Humaine et en Démographie, Cotonou, Benin; ^2^Department of Public Health, Institute of Tropical Medicine, Antwerp, Belgium; ^3^Department of Public Health and Primary Care, General Practice, Katholieke Universiteit Leuven, Leuven, Belgium; ^4^Department of Biomedical Sciences, Institute of Tropical Medicine, Antwerp, Belgium; ^5^Faculty of Health Sciences, University of Abomey-Calavi, Cotonou, Benin

**Keywords:** physicians, health workforce, primary care, COVID-19, epidemics, sub-Saharan Africa

## Abstract

**Introduction:**

In sub-Saharan Africa, there is a need to better understand and guide the practice of primary care physicians (PCPs), especially in a crisis context like the COVID-19 pandemic. This study analyses the experiences of PCPs facing COVID-19 in Benin and draws policy lessons.

**Methods:**

The study followed a fully mixed sequential dominant status design. Data were collected between April and August 2020 from a sample of PCPs in Benin. We performed descriptive analyses on the quantitative data. We also performed bivariate analyses for testing associations between various outcomes and the public/private status of the PCPs, their localization within or outside the cordon sanitaire put in place at the beginning of COVID-19, and their practice' category. A thematic content analysis was done on qualitative data. Results from both analyses were triangulated.

**Results:**

Ninety PCPs participated in the quantitative strand, and 14 in the qualitative. The median percentage of the COVID-19 control measures implemented in the health facilities, as reported by the PCPs, was 77.8% (interquartile range = 16.7%), with no difference between the various groups. While 29.4% of the PCPs reported being poorly/not capable of helping the communities to deal with COVID-19, 45.3% felt poorly/not confident in dealing with an actual case. These percentages were bigger in the private sector. The PCP's experiences were marked by anxiety and fear, with 80.2% reporting stress. Many PCPs (74.1%) reported not receiving support from local health authorities, and 75.3% felt their concerns were not adequately addressed. Both percentages were higher in the private sector. The PCPs especially complained of insufficient training, insufficient coordination, and less support to private providers than the public ones. For 72.4 and 79.3% of the PCPs, respectively, the pandemic impacted services utilization and daily work. There were negative impacts (like a decrease in the services utilization or the quality of care), but also positive ones (like improved compliance to hygiene measures and new opportunities).

**Conclusion:**

Our study highlighted the need for more structured support to PCPs for optimizing their contribution to epidemics control and good primary healthcare in Benin. Efforts in this direction can build on several good practices and opportunities.

## Introduction

Effective primary health care (PHC) has proven to improve people's health and well-being and to contribute to better and resilient health systems and societies (1, 2). While COVID-19 is straining the health systems and socio-economic structures worldwide (1), PHC is crucial to fighting the pandemic, maintaining essential health services, and preventing future crises (2, 3). However, effective PHC needs an adequate health workforce that is well-trained, sufficiently available, well-organized, well-distributed and well-performing (4).

In most sub-Saharan African countries, PHC is generally operationalized through health districts. The latter are local health systems encompassing public and private health facilities, community-based health services, alternative health services, and other support services. In a health district, the health facilities typically include first-line facilities, which are supposed to provide a large part of primary care (the service delivery component of PHC), and a district hospital (5, 6).

At many African first-line health facilities, primary care is traditionally provided by non-physicians, in most cases nurse-practitioners. However, there is a growing presence of physicians at this level of healthcare delivery, especially in the private sector (7). The existing literature shows that these primary care physicians (PCPs) have the potential to improve the quality of primary care in sub-Saharan Africa. For example, they can expand the range of services available at the primary care level and improve the technical quality of care (7). However, when their practice is not adequately prepared and regulated, as is the case in many settings, challenges can arise, such as conflicting activities with other health professionals, poor coordination, and poor performance regarding the key features of primary care (7).

These challenges may be exacerbated in a crisis context like the COVID-19 pandemic. For instance, discussions on the platform of the Global Forum on Universal Health Coverage and Primary Health suggested that, at the beginning of the pandemic, the percentages of health workers absent from work were rising, due to unsafe practices and fear to infect their family (8). In China, a qualitative study among PCPs highlighted that inadequate role definition and inadequate capacities of the PCPs were barriers to epidemic control (9). Nevertheless, as some scholars suggest, PCPs can also play a positive role in the fight against COVID-19 (10–12). Therefore, analyzing the PCP's experiences with COVID-19 can highlight opportunities for improving their practices and transforming the delivery of primary care in sub-Saharan Africa.

Previous studies on PHC and COVID-19 highlighted issues like insufficient capacities of health workers in dealing with the pandemic (13), insufficient facility preparedness (8, 13), or negative impacts of COVID-19 on health services (14–16). However, very few studies were conducted in sub-Saharan Africa (especially western and francophone Africa), and very few (8, 13, 17) looked at the specific case of PCP's experiences in contributing to the pandemic control, coping with the crisis situation, and maintaining the provision and the quality of essential primary care services.

The present study thus aims to analyze the PCP's experiences with COVID-19 in Benin, in terms of implementation of control measures recommended at the primary care level, capacity to deal with COVID-19 cases, real-life experience and support received, and impact of the pandemic on their practices. The g research questions are as follows: to what extent are the COVID-19 control measures being implemented in the health facilities where the PCPs work? To what extent do PCPs feel capable to deal with population concerns and actual COVID-19 cases? What was the psychological impact of the pandemics on PCPs and how were they supported? And finally, what is the impact of COVID-19 on PCP's practices?

The study is part of a larger research program investigating the nature and performance of the PCPs' practice in Benin to identify the most appropriate practice models.

## Materials and Methods

### Study Design

According to Leech and Onwuegbuzie's typology of mixed methods designs (18), this study follows a fully mixed sequential dominant status design (QUAN→qual). Our study is fully mixed because we mixed the quantitative and the qualitative methods at several stages of the research process, namely the sampling and the data analysis stages. The study design is sequential because the quantitative methods preceded the qualitative methods. The study has a dominant status because the quantitative data are given more weight for answering the research questions.

### Study Settings

We conducted this study in 4 of the 34 health districts in Benin: Cotonou 2 and 3 (Cot 2-3), Ouidah-Kpomassè-Tori (OKT), Parakou-N'Dali (PN) and Nikki-Kalalé-Pèrèrè (NKP). These districts are also the study sites of the broader research program on PCPs in Benin. We purposively selected them based on the geographic location to consider the socio-economic differences between the north and the south of the country and include urban and rural areas. We also considered the number of health facilities within the districts for maximizing the chance to include enough PCPs.

In Benin, the first case of COVID-19 was confirmed on 16 March 2020. As in other countries, the response strategy includes general measures (quarantine, hygiene measures or health communication) and measures to improve cases management (equipping health facilities, developing guidelines and training health workers). At the time of the quantitative data collection, there was also a *cordon sanitaire* (in place between March and May 2020), i.e., a line around the areas affected by COVID-19 (in the southern part of the country) to isolate them from the rest of the country. Cot 2-3 and OKT were situated within the borders of the cordon sanitaire. On 29 March 2021, the Minister of health launched the COVID-19 vaccination campaign in Benin. By November 2021, only 4% of the 12 million Beninese had received at least one dose of vaccine. This low coverage rate is attributed to a large extent to the population's skepticism about the safety of the COVID-19 vaccines. The Government, the health authorities and other community leaders are thus trying to sensitize the population to get vaccinated. Next to these pandemic control measures, the Government also provided social assistance to the most vulnerable populations to mitigate the socio-economic impacts of the crisis (19).

### Study Population

The study population consists of PCPs working (exclusively or not) at first-line health facilities in the four selected health districts. Following a scoping review (7) and a cross-sectional study (20), we first distinguished three categories of PCPs in Benin, based on the type of postgraduate training they received (or not):

- The category of “general practitioners” (GPs) includes PCPs without any formal postgraduate training neither on the principles of PHC or related concepts nor on any clinical speciality;
- The category of “*médecins généralistes communautaires”* (MGCs) includes physicians who attended a short postgraduate training (4 to 8 weeks) geared toward the concept and values of community-based general practice;
- The category of “specialists” refers to physicians with 4 to 5 years of postgraduate training in various clinical specialties (gynecology-obstetrics, pediatrics, rheumatology, etc.).

After analyzing their current practices (20), we further divided the category of GPs into two sub-categories: the private GPs and the public GPs, as we observed substantial differences between them. We finally obtained four categories: the public GPs, the private GPs, the MGCs, and the specialists.

### Sampling

A cross-sectional study conducted between December 2019 and July 2020 (20) identified 214 PCPs in the four districts. Among them, 150 (70%) agreed to participate in further research activities. We included all 150 PCPs in the quantitative strand, but only 90 participated (42% response rate, considering the 214 PCPs estimated in the four districts).

For the qualitative strand, we purposively selected PCPs among the 90 who participated in the quantitative strand by seeking a maximum variation regarding their model of practice and some of the most distinctive findings from the quantitative strand. The latter include the level of confidence to deal with a case, the level of stress experienced by the PCPs, and the perceived support. We reached saturation after 14 interviews.

### Data Collection

We collected quantitative data from April to May 2020 (during the first COVID-19 wave in Benin), through an online survey. The research team developed the questionnaire (Supplementary Material 1) based on the WHO guidelines for preparedness and response to COVID-19 at the primary care level (21–24). For instance, we investigated the control measures by listing the recommended measures and asking the PCPs whether they were implemented in their health facilities.

We pre-tested the questionnaire before the actual survey with five PCPs who were working outside the study sites. These PCPs were asked to fill in the questionnaire and to provide feedback. The researchers also probed for their understanding of the various questions and the need to reformulate some questions. The main recommendations from the pretests were to distinguish questions related to the provision of information to the patients from those related to the provision of information to the health providers themselves, to be more precise regarding the type of facial protection used by the health workers (respirators for high risk staff and surgical masks for the others), and to rearrange some of the questions for a smoother flow [for instance, first asking the PCPs to present the support (if any) they were receiving before investigating the specific case of training on COVID-19 management].

The questionnaire collected data on the PCP's characteristics, the different COVID-19 control measures implemented by the health facilities where they worked, the PCP's confidence level to manage a case and support the communities, the level of stress they experience, the support received, and the perceived impact of the pandemic.

We collected the qualitative data from June to August 2020 through face-to-face or phone-based semi-structured interviews. The qualitative data collection took place just after the first COVID-19 wave in Benin. The interview guide (Supplementary Material 2) explored the challenges in dealing with the pandemic and continuing to provide routine care, the nature of the pandemic's impacts, potential innovations in the organization of health services and, last but not least, lessons learnt.

The data were collected by the principal investigator (KB) and two research assistants. The principal investigator regularly assessed the quality of the data recorded by evaluating their consistency and checking their accuracy.

### Data Analysis

Quantitative data were analyzed with the Stata-MP 16.0 software. We used frequencies and percentages to describe the categorical variables and median and interquartile ranges (IQR) to describe continuous variables.

We also performed bivariate analyses to explore associations between various outcomes and the public/private status of the PCPs, their localization within or outside the cordon and their practices' category. To test these associations, we performed Fisher's exact test when the outcomes were categorical variables. When the outcome was a continuous variable, we performed the Wilcoxon rank-sum test for independent variables with two modalities and the Kruskal-Wallis rank test for independent variables with more than two modalities. A *p*-value <0.05 was considered significant.

Most of the qualitative interviews were recorded and transcribed verbatim. The data collectors immediately produced summaries of the four non-recorded interviews. We then performed a thematic content analysis supported by the Dedoose Software. This analysis started with a range of deductive themes based on quantitative results we wished to further clarify. These themes were mainly related to confirming the COVID-19 control measures implemented, the sustainability of these measures, the challenges PCPs were facing during the pandemic, the nature of the psychological impact of the pandemic on PCPs, the type of support they were receiving (or not), the nature of the COVID-19 impact on PCPs operations and service utilization, and the adaption and innovation of service delivery. These preliminary themes were further refined throughout the analysis process. The Table 1 provides an overview of the final themes yielded by the analysis.

**Table 1:** Themes and sub-themes identified during the qualitative data analysis.

**Themes**	**Sub-themes**	**Additional sub-themes**
Covid-19 control measures	Measures taken individually by the PCPs	Buying own protective equipment
		Respecting social distancing measures and hygiene measures
		Sensitizing and training the staff
	Measures taken by the health facilities	Providing some protective equipment
		Communication to the health personnel and the population
		Sometimes manufacturing their own supply (masks, hydro-alcoholic gels)
		Reinforcing the infection prevention and control measures
		Requiring use of protective equipment
		Installing handwashing devices at the entrance
		Installing isolation commodities
Coordination of the response at district level	Positive	Overall positive appreciations from the PCPs of the way the response was organized at district level
		Efforts to involve the private sector in the pandemic control activities
	Negative	Not all the providers got the right information at the same time
Capacity to manage cases and advice the population	Feeling capable of adequately informing the population	Getting good information from media and general information shared by the government
	Feeling not well prepared to deal with actual COVID-19 cases	Reporting difficulties to diagnose COVID-19 cases
		Reporting need for training on COVID-19
Challenges to deal with the pandemic	Insufficient resources	Protective equipment, financial resources
	More expenses	Buying equipment with own funds (PCPs and facilities)
		Reported more by PCPs in the private sector
	Difficulties for implementing the COVID-19 control measures in long-term	Because of resources constraints
		Because of long-term lassitude
Psychological impact of COVID-19	Stress	Decrease of usual social activities
		Need of high focus, so as not to miss a case
	Fear	Fear to get infected and to infect the family (described as “psychosis”)
		Fear to lose income
		More fear at the beginning, starting to decrease overtime
Support received from health authorities	Positive	Testing of the health workers
		Supporting the PCPs for the care for suspected and confirmed cases
		Providing information, training, equipment, testing kits, and other supplies
	Negative	Poor dialogue between health authorities and health providers, especially the private providers
		Delays from authorities in responding to calls related to suspected cases
		PCPs from the private sector feeling that the public providers were served first and better regarding training and access to resources
Impact of COVID-19 on the PCPs work	Negative impacts	Decrease of the quality of physical examination
		Additional expenses for health providers
		Reduced availability of health workers and some services
		Decrease in the incomes (for facilities and PCPs)
	Positive impacts	Improvement of communication with patients
		Improvement of the implementation of hygiene measures
		New opportunities (online courses, learning to manage epidemics)
Impact of COVID-19 on services utilization	Decrease in service utilization	Because of fear of getting infected or being put in quarantine
		Lack of financial resources to seek care
Measures for maintaining health services	Adopting new ways of working	Teleconsultations, home visits
	Easing financial access to the patients	Avoiding unnecessary care (e.g., unnecessary hospitalization)
		Being flexible in the payment modalities
Lessons learnt	COVID-19 as revelator of weaknesses	COVID-19 revealed the fragility of our health systems
	COVID-19 as a catalyst for new commitments	COVID-19 helped to understand better the necessity to comply with infection prevention and control measures

The results of the qualitative data analysis were triangulated and integrated with the quantitative results. This paper presented the quantitative and qualitative results together for a holistic description of the PCPs' experiences.

### Ethical Considerations

This study was conducted under the ethical approval N° 0193 issued by the local ethic committee for biomedical research of the University of Parakou (Benin). We obtained written consent from the participants, and we managed the data with strict confidentiality.

## Results

### Characteristics of the Primary Care Physicians

Among the 90 PCPs who responded to the survey, 70.0% were male, and the median age was 33.5 years (IQR = 12 years). The majority were practicing in urban areas (87.8%), in the private sector (85.6%), and within the boundaries of the cordon sanitaire (66.7%).

Regarding the models of practices, we categorized 7 PCPs (7.8%) as public GPs, 55 (61.1%) as private GPs, 8 (8.9%) as MGCs and 20 (22.2%) as specialists.

Among the 14 PCPs who participated in the qualitative strand, there were ten men and four women; and two public GPs, six private GPs, two MGCs and four specialists.

The Table 2 summarizes the main characteristics of the study participants.

**Table 2:** Characteristics of the study participants.

**Characteristics**	**Quantitative prong** **(*N* = 90)**	**Qualitative prong (*N* = 14)**
**Median age (IQR)**	33.5 years (IQR = 12 years)	Non applicable
**Sex**		
Male	63 (70.0%)	10
Female	27 (30.0%)	4
**Practice setting**		
Urban	79 (87.8%)	12
Rural	11 (12.2%)	2
**Institutional status**		
Public	13 (14.4%)	3
Private	77 (85.6%)	11
**Localizations within or outside the cordon sanitaire**		
Within cordon	60 (66.7%)	6
Outside cordon	30 (33.3%)	8
**PCPs category**		
Public GPs	7 (7.8%)	2
Private GPs	55 (61.1%)	6
MGCs	8 (8.9%)	2
Specialists	20 (22.2%)	4

### Implementation of the COVID-19 Control Measures Recommended at the Primary Care Level

Table 3 displays the 18 control measures assessed and the percentage of PCPs reporting the implementation of these measures in their facilities.

**Table 3:** Percentage of PCPs reporting the COVID-19 control measures in the primary care context implemented in their facilities, by institutional status and by the localization within or outside the cordon sanitaire.

	**Institutional status**			**Cordon sanitaire**			
**COVID-19 prevention and control measures**	**Public *n* (%)**	**Private** ***n* (%)**	***p*** **-value**	**Within cordon** ***n* (%)**	**Outside cordon** ***n* (%)**	***p*** **-value**	**Total** ***n* (%)**
Strengthening the cleaning of the facilities (*N* = 87)	12 (100)	75 (100)	–	58 (100)	29 (100)	–	87 (100, 0)
Strengthening measures to prevent infections during the patient's care (*N* = 86)	12 (100)	74 (100)	–	57 (100)	29 (100)	–	86 (100, 0)
Installation of a handwashing device at the facility's entrance (*N* = 86)	12 (100)	73 (98.6)	1.000	56 (98.3)	29 (100.0)	1.000	85 (98, 8)
Availability of handwashing supplies (water, soap, hydroalcoholic gel) for healthcare staff (*N* = 87)	12 (100)	73 (97.3)	1.000	56 (96.5)	29 (100.0)	0.550	85 (97, 7)
Providing COVID-19 information to patients (*N* = 88)	12 (100)	70 (92.1)	0.591	55 (94.8)	27 (90.0)	0.406	82 (93, 2)
Providing COVID-19 information to healthcare staff (*N* = 88)	12 100)	70 (92.1)	0.591	53 (91.4)	29 (96.7)	0.659	82 (93, 2)
Availability of handwashing supplies (water, soap, hydroalcoholic gel) for users (*N* = 87)	9 (75.0)	71 (94.7)	0.052	51 (87.9)	29 (100.0)	0.090	80 (92, 0)
Requiring the use of surgical masks for moderate risk staff (*N* = 87)	12 100)	64 (85.3)	0.349	53 (91.4)	23 (79.3)	0.169	76 (87, 4)
Sufficient provision of gloves (*N* = 87)	8 (66.7)	65 (86.7)	0.097	49 (84.5)	24 (82.8)	1.000	73 (83, 9)
Requiring the use of gloves for the staff involved in triage or patient care (*N* = 87)	10 (83.3)	62 (82.7)	1.000	48 (82.8)	24 (82.8)	1.000	72 (82, 8)
Requiring the use of protective face masks or respirators for staff involved in triage or patient care (*N* = 86)	6 (50.0)	50 (67.6)	0.328	37 (63.8)	19 (67.9)	0.811	56 (65, 1)
Sufficient provision of surgical masks for moderate risk staff (*N* = 87)	7 (58.3)	46 (61.3)	1.000	33 (56.9)	20 (69.0)	0.353	53 (60, 9)
Availability of a protocol for dealing with a suspected COVID-19 case (*N* = 87)	10 (83.3)	43 (57.3)	0.116	30 (51.7)	23 (79.3)	0.019^*^	53 (60, 9)
Availability of a protocol for triage (*N* = 88)	7 (58.3)	43 (56.6)	1.000	33 (56.9)	17 (56.7)	1.000	50 (56, 8)
Setting up a triage point (*N* = 88)	5 (41.7)	41 (53.9)	0.539	31 (53.5)	15 (50.0)	0.824	46 (52, 3)
COVID-19 training for the staff (*N* = 85)	6 (50.0)	35 (47.9)	1.000	23 (41.1)	18 (62.1)	0.073	41 (48, 2)
Reservation of a specific place in the facility for the consultation or isolation of suspected cases (*N* = 86)	7 (58.3)	29 (39.2)	0.227	25 (43.9)	11 (37.9)	0.649	36 (41,9)
Sufficient provision of protective face masks/respirators for staff involved in triage or patient care (*N* = 87)	2 (16.7)	28 (37.3)	0.205	17 (29.3)	13 (44.8)	0.161	30 (34,5)

* *Statistically significant at 5% level*.

The three measures most frequently reported were the strengthening of the health facility's cleaning (100%), the strengthening of measures to prevent nosocomial infections during patient care (100%) and the installation of a handwashing device at the facility's entrance (98.8%). The least reported measures were the sufficient provision of protective face masks or respirators for the staff needing them (34.5%), the reservation of a specific place in the facility for the management of suspected cases (41.9%), and the COVID-19 training for the staff (48.2%). The qualitative interviews confirmed these results and revealed some coping measures from the physicians. For instance, PCPs reported that they bought protective equipment themselves.

For most COVID-19 control measures, we found no significant difference in the percentage of PCPs reporting them, according to their institutional status (public or private) or whether the facilities were located within or outside the cordon sanitaire. However, the percentage of PCPs reporting the availability of a protocol for dealing with a case appears to be higher outside the cordon (*p* = 0.019, Table 3).

We found no significant difference in the percentage of PCPs reporting the implementation of control measures, depending on their category (Supplementary Material 3).

Analyzing all measures together, the median percentage of measures reported by PCPs was 77.8% (IQR = 16.7%). There was no statistically significant difference related to public or private status, location within or outside the cordon, or PCPs' category (Supplementary Materials 4, 5).

### PCPs' Perceived Capacity to Manage COVID-19

Regarding the PCP's ability to deal with COVID-19, 29.4% of them felt they were poorly or not capable of helping the communities deal with COVID-19 (e.g., through counseling). A higher proportion (45.3%) felt poorly or not confident in dealing with an actual COVID-19 case (Table 4).

**Table 4:** Perceived capacity, confidence, level of stress, support received and impact of COVID-19, by institutional status and by the localization within or outside the cordon sanitaire.

	**Institutional status**			**Cordon sanitaire**			**Total**
**Various outcomes**	**Public *n* (%)**	**Private** ***n* (%)**	***p-*** **value**	**Within cordon** ***n* (%)**	**Outside cordon *n* (%)**	***p*** **-value**	
**Perceived capacity to support the communities in dealing with COVID-19 (*****N*** **= 85)**							
Highly capable	7 (58.3)	14 (19.2)		12 (21.4)	9 (31.0)		21 (24.7)
Moderately capable	2 (16.7)	37 (50.7)	0.014^*^	26 (46.4)	13 (44.8)	0.611	39 (45.9)
Poorly or not capable	3 (25.0)	22 (30.1)		18 (32.1)	7 (24.1)		25 (29.4)
**Level of confidence to deal with a suspected COVID-19 case (*****N*** **= 86)**							
Highly confident	6 (50.0)	11 (14.9)		9 (15.8)	8 (27.6)		17 (19.8)
Moderately confident	1 (8.3)	29 (39.2)	0.014^*^	23 (40.4)	7 (24.1)	0.239	30 (34.9)
Poorly or not confident	5 (41.7)	34 (45.9)		25 (43.9)	14 (48.3)		39 (45.3)
**Increase of stress (*****N*** **= 86)**							
High	1 (8.3)	20 (27.0)		17 (29.8)	4 (13.8)		21 (24.4)
Moderate	8 (66.7)	40 (54.1)	0.371	27 (47.4)	21 (72.4)	0.101	48 (55.8)
No increase	3 (25.0)	14 (18.9)		13 (22.8)	4 (13.8)		17 (19.8)
**Support received from local health authorities (*****N*** **= 85)**							
Received support	8 (66.7)	14 (19.2)	0.002^*^	11 (19.6)	11 (37.9)	0.115	22 (25.9)
Have not received support	4 (33.3)	59 (80.8)		45 (80.4)	18 (62.1)		63 (74.1)
**Degree to which worries and concerns were addressed by local health authorities (*****N*** **= 85)**							
Fully addressed	2 (16.7)	1 (1.4)		1 (1.8)	2 (6.9)		3 (3.5)
Moderately addressed	4 (33.3)	14 (19.2)	0.019^*^	8 (14.3)	10 (34.5)	0.027^*^	18 (21.2)
Poorly or not addressed	6 (50.0)	58 (79.5)		47 (83.9)	17 (58.6)		64 (75.3)
**Perceived impact on PCP's daily work (*****N*** **= 87)**							
High impact	7 (58.3)	30 (40.0)		30 (51.7)	7 (24.1)		37 (42.5)
Moderate impact	2 (16.7)	30 (40.0)	0.307	19 (32.8)	13 (44.8)	0.037^*^	32 (36.8)
Little or no impact	3 (25.0)	15 (20.0)		9 (15.5)	9 (31.0)		18 (20.7)
**Perceived impact on services utilization (*****N*** **= 87)**							
High impact	4 (33.3)	30 (40.0)		31 (53.4)	3 (10.3)		34 (39.1)
Moderate impact	3 (25.0)	26 (34.7)	0.535	16 (27.6)	13 (44.8)	<0.001^*^	29 (33.3)
Little or no impact	5 (41.7)	19 (25.3)		11 (19.0)	13 (44.8)		24 (27.6)

* *Statistically significant at 5% level*.

The qualitative data showed that PCPs had general information on COVID-19, but they did not feel adequately trained for dealing with cases or implementing specific infection prevention and control measures:

“*We work with the knowledge we have. We have not yet been really prepared for this. I mean, to continue to manage other pathologies while thinking about limiting the spread of COVID-19. For example, in the maternity ward, when women come to give birth, we must integrate COVID-19's prevention into their care. But we need to find knowledge or other training so that we know how to integrate covid-19 prevention into other activities.”* PCP, NKP.

We found no significant difference in the PCPs' perceived capacity to support the communities or their confidence level to deal with a case according to their localization within or outside the cordon (Table 4) or according to their categories (Table 5). However, there seems to be a higher percentage of private PCPs (compared to the public ones) that felt poorly or not capable of supporting the communities or poorly or not confident to deal with a case (*p* = 0.014 for both variables, Table 4).

**Table 5:** Perceived capacity, confidence, level of stress, support received and impact of COVID-19, by the PCPs' categories.

	**PCP's category**					
**Various outcomes**	**Public GPs** ***n* (%)**	**Private GPs** ***n* (%)**	**MGCs** ***n* (%)**	**Specialists** ***n* (%)**	***p*** **-value**	**Total** ***n* (%)**
**Perceived capacity to support the communities in dealing with COVID-19 (*****N*** **= 85)**						
Highly capable	4 (66.7)	9 (17.3)	2 (28.6)	6 (30.0)		21 (24.7)
Moderately capable	0 (0.0)	26 (50.0)	3 (42.9)	10 (50.0)	0.111	39 (45.9)
Poorly or not capable	2 (33.3)	17 (32.7)	2 (28.6)	4 (20.0)		25 (29.4)
**Level of confidence to deal with a suspected COVID-19 case (*****N*** **= 86)**						
Highly confident	3 (50.0)	7 (13.2)	2 (28.6)	5 (25.0)		17 (19.8)
Moderately confident	1 (16.7)	20 (37.7)	3 (42.9)	6 (30.0)	0.390	30 (34.9)
Poorly or not confident	2 (33.3)	26 (49.1)	2 (28.6)	9 (45.0)		39 (45.3)
**Increase of stress (*****N*** **= 86)**						
High	0 (0.0)	13 (24.5)	2 (28.6)	6 (30.0)		21 (24.4)
Moderate	5 (83.3)	29 (54.7)	5 (71.4)	9 (45.0)	0.605	48 (55.8)
No increase	1 (16.7)	11 (20.8)	0 (0.0)	5 (25.0)		17 (19.8)
**Support received from local health authorities (*****N*** **= 85)**						
Received support	4 (66.7)	13 (24.5)	1 (14.3)	4 (21.1)	0.141	22 (25.9)
Have not received support	2 (33.3)	40 (75.5)	6 (85.7)	15 (78.9)		63 (74.1)
**Degree to which worries and concerns were addressed by local health authorities (*****N*** **= 85)**						
Fully addressed	2 (33.3)	0 (0.0)	0 (0.0)	1 (5.0)		3 (3.5)
Moderately addressed	1 (16.7)	12 (23.1)	3 (42.9)	2 (10.0)	0.017^*^	18 (21.2)
Poorly or not addressed	3 (50.0)	40 (76.9)	4 (57.1)	17 (85.0)		64 (75.3)
**Perceived impact on PCP's daily work (*****N*** **= 87)**						
High impact	3 (50.0)	20 (36.4)	1 (16.7)	13 (65.0)		37 (42.5)
Moderate impact	1 (16.7)	25 (45.5)	1 (16.7)	5 (25.0)	0.030^*^	32 (36.8)
Little or no impact	2 (33.3)	10 (18.2)	4 (66.7)	2 (10.0)		18 (20.7)
**Perceived impact on services utilization (*****N*** **= 87)**						
High impact	3 (50.0)	21 (38.2)	0 (0.0)	10 (50.0)		34 (39.1)
Moderate impact	1 (16.7)	22 (40.0)	2 (33.3)	4 (20.0)	0.122	29 (33.3)
Little or no impact	2 (33.3)	12 (21.8)	4 (66.7)	6 (30.0)		24 (27.6)

* *Statistically significant at 5% level*.

### Real-Life Experiences and Support Received

Most PCPs (80.2%) reported a moderate or high increase in stress due to the pandemic. There was no significant difference between the various groups of PCPs (Tables 4, 5).

Stress and fear were also recurrent themes in the discourse of the PCPs interviewed. Many physicians related this to the fear of getting infected, but some mentioned the fear of losing income (especially in private practices) and the fear of misdiagnosing a suspect case.

“*It was total chaos. Everyone was scared, doctors and nurses alike. People suggested to me to close this clinic, for example. The staff then said they were going to stay at home. It was a very difficult time of fear, panic. The staff were even running away from the patients... It is now that the pressure has started to ease.”* PCP, Cot 2-3.

As for the support received, 74.1% of the PCPs reported not receiving support from the local district health managers. This percentage appeared higher in the private sector than in the public sector (*p* = 0.002). There were no statistically significant differences for this variable according to the localization within or outside the cordon or according to the PCP's category (Tables 4, 5). For the PCPs who indicated to have received support, the latter included training, offering equipment and diagnostic kits, and providing orientations to manage cases.

Among the PCPs, 75.3% stated that their concerns and worries were poorly or not addressed by the local health authorities. This proportion appeared higher among the PCPs in the private sector (*p* = 0.019) and those within the cordon sanitaire (*p* = 0.027, Table 4). We also noticed a statistically significant difference between various categories of PCPs, with more specialists reporting that their concerns were not addressed, followed by the private GPs (*p* = 0.017, Table 5).

The qualitative prong also highlighted differences between public and private PCPs in the support received. Many private PCPs complained of several issues: delays receiving the correct information, insufficient support in getting the protective equipment, delays in responding to the requests related to a suspected case, insufficient training, and insufficient coordination.

“*For coordination… hum… in my district, I think there is a real information problem. The information comes to the district managers, okay. Does the information come at the facilities' level? Because I work in three different clinics, I should normally be informed if there is information coming from the district management. Unfortunately, the few examples where they provide support are mainly related to case management or screening. We call them when we have suspected cases. We call the COVID centre that will designate the health district or a doctor. I don't know how it is organized... So, is it well coordinated? Well, anyway, when I call them, it takes a while for them to come. But they always end up responding somehow.”* PCP, Cot 2-3.

Many private PCPs felt that the public health staff were better served in terms of in-service training and other resources. Some of them even said that they lost confidence in the health system. The following verbatim provides an example illustrating this particular issue.

“*Apart from COVID-19, you need continuous training in relation to (other) pathologies. That is what is lacking in our country, because for all (training) that comes in, it is the public (providers) that they care for first. And when they finish with the public agents, it is perhaps now that they remember those who are in the private sector.”* PCP, NKP.

### Impact of COVID-19 on the PCPs' Work and Services Utilization

COVID-19 had a high or moderate impact on health services utilization, as reported by 72.4% of the PCPs (Tables 4, 5). Such an impact was more frequently reported by PCPs operating within the cordon sanitaire (*p* < 0.001, Table 4). There was no difference for this variable according to the PCP's public or private status or according to their category.

For the PCPs interviewed, this impact was mainly a decrease in the utilization of the services:

“*The health centres are almost empty now. Before, we saw 15 to 20 patients a day, and now we see about 10 a day.”* PCP, Cot 2-3.

Two main themes emerged when we explored the reasons for the decline in utilization: fear and financial barriers. For most PCPs, people were afraid to go to health facilities, either by fear of contracting COVID-19 or of being isolated and stigmatized when they had COVID-like symptoms.

“*People don't want to come because they are afraid. They think they are going to get the coronavirus by coming to the health centre... The dispensaries are the most affected. It is in these services that people go when they feel a respiratory illness. So, when the symptoms are a little bit like the coronavirus symptoms, it's a panic, you know? They are afraid to go to the hospital. They imagine people will suspect them...”* PCP, NKP.

Regarding financial barriers, the doctors stated that many people have seen their economic activities slow down because of the closure of borders and other pandemic control measures.

“*Because of COVID-19, there are many who saw their business drop. Some used to get goods from foreign countries for their business, but now they cannot cross the borders. So, if they want to come to the hospital, they cannot afford it. People who used to pay properly, when you treat them now, they say they don't have money.”* PCP, NKP.

Several respondents suspected that the decrease in service utilization had negative consequences such as increased self-medication and delays in receiving adequate treatment.

Most PCPs (79.3%) felt that COVID-19 highly or moderately impacted their daily work. This percentage seemed higher among the PCPs within the cordon sanitaire (*p* = 0.037, Table 4). The specialists had the highest percentage of physicians reporting an impact on their work, followed by the private GPs (*p* = 0.030, Table 5). We did not find any significant difference according to the public or private status (Table 4).

From the qualitative data, we noticed both negative and positive impacts on PCPs' work. On the negative side, there was potentially a limitation of the availability of the services. Some facilities had to limit the number of patients to be seen per day or postpone some activities (family planning, for instance). Also, the interviews highlighted a decrease in the availability of health workers during the peak of the pandemic. The health workers were sometimes absent because of the fear of getting infected. The PCPs also reported staff reduction in the health facilities to cope with the financial consequences of the crisis.

“*There is a staff reduction in a health facility where I was working. I lost my shifts in this facility, which impacted my income.”* PCP, Cot 2-3.

The above quote also indicates that the pandemic has had financial consequences for both PCPs and the facilities where they work. Unlike the quantitative data, the qualitative analysis suggested that the financial impact of the crisis was more pronounced in the private sector, especially because of additional expenses for the COVID-19 response and reduced service utilization.

“*Well, we have financial difficulties... The decrease in attendance has led to a drop in income for us... The payment of the staff has become difficult...”* PCP, Cot 2-3.

Another negative impact was the decrease in the quality of the physical examination and the risk of diagnostic errors:

“*... now, any fever is first taken as COVID-19 when the patient may be having pneumonia. It would be wise for the doctor to take all the protective measures and do what he needs to do so that he doesn't miss the diagnosis. Because when you are suspected of having COVID-19, there is this fear of approaching you. You could be suffering from pneumonia, severe malaria, or any other disease with flu-like symptoms; you are no longer examined.”* PCP, Cot 2-3.

PCPs also reported positive impacts from COVID-19. Almost all of them testified they had improved compliance with hygiene and infection control measures, as showed by the quote below (and also Table 3):

“*If we knew before that we should respect hygiene measures, COVID-19 has further accentuated our compliance. It has led us to protect ourselves better when we are consulting (patients). I think even after COVID-19, these habits can't go away.”* PCP, PN.

PCPs also noted that they were communicating better with their patients because they needed to educate and reassure them about COVID-19.

“*COVID-19 has really come to teach us a new way of being. For me, it taught me to communicate. I spend all my time communicating.”* PCP, NKP.

Furthermore, PCPs had to find strategies to mitigate the impact of COVID-19 on service utilization. Several practiced teleconsultations by interacting with the patients through phone calls or WhatsApp and deciding on appropriate action. To reduce financial barriers, some PCPs reported reducing the costs of consultations. Others reported that they try to avoid unnecessary care for reducing costs and the risk of contamination.

Finally, PCPs emphasized that COVID-19 had opened new opportunities, such as better awareness and access to e-learning, the use of remote consultations, and the opportunity to learn more about epidemic management.

The main lesson learnt by PCPs during this pandemic was the need to improve the health system and be better prepared for future outbreaks:

“*We need to rethink the basics of our health care system, establish very good protocols for care...There will probably be other epidemics.”* PCP, OKT.

## Discussion

### Main Results and Comparison With International Literature

The median percentage of COVID-19 measures implemented in the health facilities, as reported by the PCPs, was 77.8% (IQR = 16.7%), with no difference between the various groups. It is encouraging to have half of the PCPs reporting that their facilities implemented more than three-quarters of the COVID-19 control measures. However, key control measures were poorly implemented, such as health workers training or sufficient provision of protective face masks. Other studies, conducted in the second and third quarters of 2020 as ours, also found a deficit in these measures (13, 25). A study from primary care settings in several continents indicated a poor preparation for managing the pandemic (8), at least at the beginning.

Interestingly, we did not find significant differences between the public and the private sectors in implementing the COVID-19 control measures. Indeed, private health providers in Benin (and elsewhere in Africa) are often criticized for not properly following the existing guidelines (7, 26). Literature from low- and middle-income countries also indicates that private providers comply less with medical standards of practice than public providers do (27). However, our study showed that private PCPs implemented three quarters of the COVID-19 control measures (Supplementary Material 4), and that they performed as well as public PCPs. Even if we cannot draw definitive conclusions based on this single study, other scholars also reported that the private sector has contributed to fighting the pandemic (14, 28).

Unexpectedly, more PCPs outside the *cordon sanitaire* (i.e., an area with low COVID-19 transmission rates at the time of data collection) than inside reported that the health managers provided a protocol for dealing with suspected cases. This may indicate that information flowed equally in both areas, thanks to communication technologies. It may also be linked to confounding factors. For example, it has been documented that the performance of health facilities (including private ones) can be impacted by the leadership and the performance of local health managers (29). Therefore, some health managers outside the cordon sanitaire may have been more proactive than others in distributing protocols and t mobilizing health care providers.

In our study, 29.4% of the PCPs reported being poorly or not capable of helping the communities to deal with COVID-19. In addition, almost half (45.3%) of the PCPs felt poorly or not confident in dealing with an actual case. In contrast, a study in Uganda reported that most health workers felt confident to manage a COVID-19 case (30). Based on the qualitative data and the assessment of control measures, we can explain our results by insufficient training and poor availability of protective equipment. Indeed, the Ugandan study (30) and a literature review (17) found a positive correlation between the level of knowledge and the health worker's attitudes. Another possible explanation of our results is that the data collection took place between March and August 2020, at the very beginning of the COVID-19 pandemic in Benin. At that time, PCPs and other health workers were not yet familiar with the disease, and response strategies were still being refined, even at the national level. Based on *ad hoc* field observations, it is likely that the situation evolved since, with a better capacity of PCPs to support communities and properly manage COVID-19 cases. The Ugandan study cited above was conducted at a later stage, between September and November 2020 (30). This could explain the higher confidence level observed among health workers. Nevertheless, our results call for better preparation of PCPs (and other primary care workers for that matter) to deal with epidemics before they occur.

The percentage of PCPs who felt poorly or not capable of supporting the communities or poorly or not confident in dealing with a case appeared higher among the private PCPs than among the public ones. Although we cannot exclude confounding factors, this finding raises the question of the access to in-service training provided for private PCPs by Beninese health authorities. Previous studies (7) and some qualitative results of our study (discussed below) also stressed the need for better access to training and other resources in the private sector.

The majority of PCPs (80.2%) reported increased personal stress with the pandemic. The qualitative data amply illustrated this stress and the fear of PCPs and other health workers, especially at the beginning of the pandemic. Several literature reviews (31–33) and a study in 13 African countries (13) also reported high levels of stress among health workers during COVID-19 and other epidemics. This stress was related to the fear of being infected and infecting one's family, inadequate protective equipment, increased workload, and feelings of inadequate preparation (31–33). Our study confirmed these factors.

Adequate support of health workers is strongly recommended during pandemics, and many countries (mainly in high-income settings) adopted measures in this regard in the first months following the start of the pandemic (34, 35). However, our results indicate insufficient support to PCPs in Benin at the time of data collection, particularly concerning the provision of timely information, the acquisition of the necessary equipment for the COVID-19 response and for the handling of the financial and psychological hardships resulting from the pandemic. Indeed, 74.1% of the PCPs reported not having received any support from the local health authorities, especially in the private sector. Similarly, 75.3% of the PCPs felt that their concerns were not properly addressed by the health authorities, with a higher percentage for PCPs in the private sector and those located within the cordon sanitaire. Other studies confirmed the inadequate support during epidemics for health workers in developing countries (31–33). A study conducted in July and August 2020 in eight African countries also highlighted the insufficient support to private health workers (14, 28). Moreover, the categories of specialists and private GPs seemed to have higher percentages of unsatisfied PCPs than the two others. This may be because most of them are in the private sector, with usually little support from the State (7, 14).

For 72.4% of the PCPs, COVID-19 impacted health services utilization, especially within the cordon sanitaire. This impact was mainly a decrease in utilization because of fear and financial barriers. Although we could not confirm the doctors' declarations with observations, our results are consistent with those of a systematic review that found a median reduction of 37% in service utilization in several countries (16).

Many PCPs (79.3%) also reported that the pandemic impacted their daily work. This impact was most reported within the cordon sanitaire and among the PCPs belonging to the specialists and the private GP's categories. Moreover, even though there was no statistically significant difference between the public and the private sectors for this variable, the qualitative data highlighted that the private PCPs and their facilities faced significant financial impacts. Another study also reported the financial impacts of COVID-19 on private health facilities (14).

Finally, despite these negative impacts (14, 34, 36), PCPs also reported positive impacts such as improved compliance with hygiene measures, better communication with patients, and adopting new and innovative ways to work. We also found similar good practices in the literature (8, 13, 34, 35).

### Implications for Primary Health Care

Despite the hopes brought by vaccination, COVID-19 is not yet behind us, and other pandemics may occur (36, 37). Our study highlighted insufficient preparation and a significant impact of the pandemic at the primary care level. Moreover, the recurrent dysfunctions of many African health systems appear to be exacerbated. For example, we found a decrease in the quality of care, which was already an issue in our settings (7, 38). The insufficient collaboration between the private providers and health authorities, the lack of a structured mechanism to support PCPs or the insufficient coordination of health actors at the district level (7) are also dysfunctions that existed before the crisis.

Therefore, the effective containment of COVID-19 and future epidemics must include determined efforts to restructure African health systems and make them more effective, adaptative, and resilient. This restructuration will also help avoid the interruption of essential services (37, 39).

Despite all the shortcomings noted by this study, it also highlighted good practices and opportunities that can support this restructuring. First, even if their response was not perfect and despite limited resources, the PCPs demonstrated an appreciable capacity to react promptly to the pandemics and innovate. Like in other settings (8, 13, 34, 35), they adapted their practice to fight the pandemic and mitigate its impact. Secondly, our study found that private providers made real efforts to contribute to the response. Their performance in the pandemic response appears similar to that of the public providers. This finding suggests that the private providers can, under certain conditions, serve a public goal, as described by some authors (40, 41). This could encourage health authorities to better collaborate with private providers in epidemic control. Finally, the pandemic offers a window of opportunity to introduce changes at the primary care level. Our results indicate that PCPs are more aware of the importance of some practices, such as communicating with patients or complying with hygiene measures. PCPs also reported increased use of technology, which offers new ways for learning and working. These positive aspects open new perspectives and constitute levers for action to strengthen primary care delivery in Benin and even in sub-Saharan Africa.

### Future Research Needs

Future research can deepen the knowledge of how PCPs manage COVID-19 and how the primary care level is equipped to respond to pandemics. A first example is to check whether the good practices and the COVID-19 control measures implemented last over time. Secondly, our health systems would benefit from systematic documentation of epidemic preparedness and its impact on the delivery and utilization of essential health services. Finally, health managers and PCPs may design and test evidence-based interventions to strengthen primary care, and better prepare primary care providers for health crises in Benin and, more generally, in sub-Saharan Africa. Our findings can inform the design of such interventions.

### Strengths and Limitations

We followed a rigorous methodology to design the research protocol, develop the tools, collect and manage the data. The study also benefited from the inherent strengths of mixed methods, namely the ability to triangulate data and analyze the same phenomenon from multiple perspectives (42).

However, there are some limitations. For the quantitative prong, there may be a selection bias related to non-respondents. The main reason given by non-respondents was lack of time. However, the characteristics of the respondents were similar to those of the target population (20). The cross-sectional design of our study did not allow us to capture changes over time. Because of this limitation and given the fact that data was collected at the very beginning of the pandemic in Benin, it is likely that the situation described in this paper evolved over time. Therefore, our conclusions focused on lessons learnt for improving epidemic preparation and primary care in the future rather than judging the current preparedness status. Finally, there could be an information bias, as the study was based on self-reporting by PCPs which may have led to socially desirable answers (43). We strived to mitigate this risk of bias through several techniques. First, during data collection, we emphasized that the study aimed at learning (and not at blaming individual PCPs). Moreover, we carefully conducted the qualitative interviews and triangulated the data. We also welcomed feedback on the results from PCPs and health authorities.

## Conclusion

This study adds to the body of knowledge on the PCPs' physicians practices in sub-Saharan Africa and how they deal with the current COVID-19 pandemics. The findings highlighted the importance of improving the preparation of PCPs to deal with epidemics, the need to support them better, and the need for a better engagement of the private sector. Finally, this research found that the crisis did not only have adverse effects. It also revealed strengths and opportunities that could be exploited to improve the health system sustainably.

## Data Availability Statement

The raw data supporting the conclusions of this article will be made available by the authors, without undue reservation.

## Ethics Statement

The studies involving human participants were reviewed and approved by the Local Ethics Committee for Biomedical Research, University of Parakou, Benin. The patients/participants provided their written informed consent to participate in this study.

## Author Contributions

KB co-conceptualized the study, coordinated the data collection, curated the data, performed quantitative and qualitative data analysis, and produced the first draft of the manuscript. JDL supervised the research process, contributed to the qualitative data analysis, and reviewed the manuscript drafts. CA contributed to the data collection, the data curation, the quantitative data analysis, and reviewed the manuscript drafts. DMZ supervised the research process and reviewed the manuscript drafts. LA provided methodological inputs and reviewed the manuscript drafts. BC co-conceptualized the study, supervised the research process, contributed to the quantitative and the qualitative data analysis, and reviewed the manuscript drafts. All authors have read and approved the manuscript.

## Funding

The authors received a grant of 5,000 Euros from the Department of Public Health of the Institute of Tropical Medicine Antwerp as part of the institute's efforts to combat COVID-19. The department did not influence the research questions or the result's orientation.

## Conflict of Interest

The authors declare that the research was conducted in the absence of any commercial or financial relationships that could be construed as a potential conflict of interest.

## Publisher's Note

All claims expressed in this article are solely those of the authors and do not necessarily represent those of their affiliated organizations, or those of the publisher, the editors and the reviewers. Any product that may be evaluated in this article, or claim that may be made by its manufacturer, is not guaranteed or endorsed by the publisher.

## Abbreviations

Cot 2-3: Cotonou 2 and 3
GP: General practitioner
IQR: Interquartile range
MGC: Médecin généraliste communautaire
NKP: Nikki-Kalalé-Pèrèrè
OKT: Ouidah-Kpomassè-Tori
PN: Parakou-N'Dali
PCP: Primary care physician
PHC: Primary health care.
