# Supplementary material for: Lessons Learnt From the Experiences of Primary Care Physicians Facing COVID-19 in Benin: A Mixed-Methods Study
Source: Front Health Serv. 2022 Mar 29;2:843058. doi: 10.3389/frhs.2022.843058 (PMC10012796; doi:10.3389/frhs.2022.843058)
Supplement: Supplementary file 1 [file Table_1.docx]

Supplementary Material 1

**Covid-19: Experience of Primary Care Physicians in Benin**

Questionnaire

1. **Identification of the respondent**

| **Q1** | Please, report the confidential code you receive by email |
| --- | --- |
| **Q2** | In which commune is the health facility where you currently work, most of the time? *(Choose the name of the commune)*   1. Cotonou 2. Ouidah 3. Tori-Bossito 4. Kpomassè 5. Nikki 6. Kalalé 7. Pèrèrè 8. Parakou 9. N'Dali 10. Other (specify) |
| **Q3** | What is your sex? *(Choose the corresponding answer)*   1. Male 2. Female |
| **Q4** | How old are you (in years)? *(Report the answer)* |
| **Q5** | What is your current speciality? *(Choose the corresponding answer)*   1. General practitioner 2. *"Médecin généraliste Communautaire"* 3. Clinical specialist 4. Public Health Specialist 5. Other (Specify) |

1. **Preparation and response to COVID-19 in your health facility**

| **Q6:** | Has your health facility taken the following measures to control COVID-19?  *(Choose the corresponding answer for each measure)* |
| --- | --- |
| **a** | Awareness posters or distribution of brochures on COVID-19 for patients and accompanying persons   1. Yes 2. No 3. I don’t know |
| **b** | Information and education sessions on COVID-19 for patients and accompanying persons   1. Yes 2. No 3. I don’t know |
| **c** | Other communication measures for patients and accompanying persons   1. Yes (please, describe) 2. No 3. I don’t know |
| **d** | Posters, brochures, or other materials to inform and raise awareness among healthcare providers   1. Yes 2. No 3. I don’t know |
| **e** | Information and/or training sessions on COVID-19 for healthcare providers   1. Yes 2. No 3. I don’t know |
| **f** | Other communication measures for healthcare providers   1. Yes (please, describe) 2. No 3. I don’t know |
| **g** | Existence of a clear protocol for the triage and orientation of patients received in the health facility   1. Yes 2. No 3. I don’t know |
| **h** | Setting up a triage point for patients to detect suspected COVID-19 cases on arrival at the facility?   1. Yes 2. No 3. I don’t know |
| **i** | Reservation of a specific room or area for waiting, consultation and/or isolation of suspected covid-19?   1. Yes 2. No 3. I don’t know |
| **j** | Installation of a handwashing device at the entrance to the health facility?   1. Yes 2. No 3. I don’t know |
| **k** | Requiring the use of gloves by the staff involved in triage or patient care   1. Yes 2. No 3. I don’t know |
| **l** | Requiring the use of protective face masks (N95, FFP2, FFP3) or respirators for staff involved in triage or patient care   1. Yes 2. No 3. I don’t know |
| **m** | Requiring the use of surgical masks for moderate risk staff (drivers, reception staff apart from those at the triage point, etc.)?   1. Yes 2. No 3. I don’t know |

|  |  |
| --- | --- |
| **Q7** | Have the general infection prevention measures implemented by the staff been reinforced because of COVID-19? *(e.g., hand washing before and after each patient care, use of gloves for invasive procedures, etc., choose the corresponding answer)*   1. Yes, they have been reinforced 2. No, nothing changed 3. No, it is worse than before   I don’t know |
| **Q8** | Has the routine cleaning of the health facility been reinforced due to covid-19? *(Choose the corresponding answer)*   1. Yes, it has been reinforced 2. No, nothing changed 3. No, it is worse than before 4. I don’t know |
| **Q9** | To what extent are gloves available to staff in your health facility to prevent COVID-19 infection? *(Choose the appropriate answer based on your own experience)*   1. Sufficiently available 2. Moderately available 3. Poorly available 4. Not available at all |
| **Q10** | To what extent are face masks (N95, FFP2, FFP3) or respirators available for the staff responsible for triage or patient care in your health facility for the prevention of COVID-19 infection? *(Choose the appropriate answer based on your own experience)*   1. Sufficiently available 2. Moderately available 3. Poorly available 4. Not available at all available |
| **Q10-1** | To what extent are surgical masks available for the moderate-risk staff in your health facility for the prevention of COVID-19 infection? *(Choose the appropriate answer based on your own experience)*   1. Sufficiently available 2. Moderately available 3. Poorly 4. Not available at all |
| **Q11** | To what extent are handwashing supplies (water+ soap or hydroalcoholic gel) available **for the staff**? *(Choose the appropriate answer based on your own experience)*   1. Sufficiently available 2. Moderately available 3. Poorly available 4. Not available at all |
| **Q12** | To what extent are handwashing supplies (water+ soap or hydroalcoholic gel) available **for the patients and accompanying persons**? *(Choose the appropriate answer)*   1. Sufficiently available 2. Moderately available 3. Poorly available 4. Not available at all |
| **Q13** | Has your health facility or the health district provided you with a protocol for dealing with a suspected case of COVID-19 and procedures for referral to the appropriate management site? *(Choose the corresponding answer)*   1. Yes 2. No |
| **Q14** | Currently, to what extent do you feel confident for receiving and orienting a patient suspected of having COVID-19 if you receive one in your health facility? *(Choose the corresponding answer)*   1. Highly confident 2. Moderately confident 3. Poorly confident 4. Not confident at all   What are the reasons for your answer? |
| **Q15** | Have you ever received a case of COVID-19?   1. Yes 2. No   If yes, what were your main difficulties in dealing with the case(s)? |

3. Support you are receiving during the COVID 19 pandemic

| **Q16** | Are you receiving or have you received support from the health district management team for the COVID-19 epidemic? *(Choose the corresponding answer)*   1. Yes 2. No |
| --- | --- |
| **Q16-1** | (If yes) List below the support received |
| **Q17** | Have you received training/refresher training on COVID-19? *(Choose the corresponding answer)*   1. Yes 2. No |
| **Q17-1** | If yes, by which institution was the training provided? *(Select all that apply)*   1. Your health facility 2. The health district office 3. The Departmental Health Directorate 4. The Ministry of Health 5. An NGO or other civil society organisation 6. Other (please specify) |
| **Q17-2** | If yes, what were the main themes of this training/refresher training? *(Select all that apply)*   1. Preventive measures against COVID-19 2. General infection prevention and control measures in health care settings 3. Use of personal protective equipment (gloves, masks, gown, glasses, etc.) 4. Handwashing 5. Triage and case detection 6. Management of suspected, probable, or confirmed cases 7. Other (please specify) |
| **Q 18** | How would you rate your stress level at present? *(Choose the corresponding answer)*   1. As usual 2. Moderately higher than usual 3. Much higher than usual |
| **Q 19** | To what extent would you say that the health district's authorities have adequately addressed your concerns? *(Choose the corresponding answer)*   1. Fully addressed 2. Moderately addressed 3. Poorly addressed 4. Not addressed at all |

1. **Experience of the COVID-19 pandemic by the population**

| **Q20** | From your experience, how is the population (your patients and the rest of the community) experiencing this period of pandemic? |
| --- | --- |
| **Q21** | To what extent do you feel capable of adequately supporting the population through this crisis? *(Choose the corresponding answer)*   1. Highly capable 2. Moderately capable 3. Poorly capable 4. Not capable at all   What are the reasons for your answer? |

1. **Impact of COVID 19 on your work and the utilisation of services**

| **Q 22** | To what extent has the current Covid-19 epidemic impacted your daily work? *(Choose the corresponding answer)*   1. High impact 2. Moderate impact 3. Little impact 4. No impact at all | |
| --- | --- | --- |
| **Q 22-1** | Please describe the impact that COVID-19 has had on your daily work. | |
| **Q 23** | To what extent has the current Covid-19 pandemic affected the utilisation of your services by patients? *(Choose the corresponding answer)*   1. High impact 2. Moderate impact 3. Little impact 4. No impact at all |  |
| **Q 23-1** | Please describe the impact that COVID-19 has had on the utilisation of your services by the patients. |  |
| **Q 24** | Are there any measures implemented by yourself or within your health facility to continue to provide effectively essential healthcare to patients despite the COVID-19 pandemic? *(Choose the appropriate answer)*   1. Yes 2. No   If yes, describe these measures. |  |
| **Q 25** | Which measures would you like to see implemented to support you in continuing to provide essential healthcare services to your patients? |  |
