# Supplementary material for: Lessons Learnt From the Experiences of Primary Care Physicians Facing COVID-19 in Benin: A Mixed-Methods Study
Source: Front Health Serv. 2022 Mar 29;2:843058. doi: 10.3389/frhs.2022.843058 (PMC10012796; doi:10.3389/frhs.2022.843058)
Supplement: Supplementary file 2 [file Table_2.DOCX]

Supplementary Material 2

**Covid-19: Experience of Primary Care Physicians in Benin**

Interview guide for primary care physicians

Instruction to the interviewer

# Method for conducting the interview

You can conduct the interview in three ways:

- Zoom call (create a zoom link and send it to the physician via WhatsApp or email)
- Direct phone call
- Visit the physician for a face-to-face interview (make sure the COVID-19 measures are strictly respected)

# Introduction:

- Introduce yourself (name, qualification, institution)
- Explain the objective of the interview (i.e., to deepen the understanding of the experience of the primary care physicians facing COVID-19 in Benin, to understand better the impact of the pandemic on the functioning of health services, and to know the primary care physicians’ needs for coping with the pandemic and continuing the provision of essential health services)
- Guarantee confidentiality and anonymisation of the data
- Ensure that the respondent sign the informed consent sheet
- Ask permission to record the interview and take notes.
- Report the physician’s confidential code on the notes
- Report the start time of the interview:
- Note the interview end time:
- Properly label the record using the confidential code of the physician.

# Interview questions

## Part 1: Appreciation of the primary care physicians’ experience in responding to COVID-19

- Please, tell us about the COVID-19’s response:
  - At the national level
- (follow-up question) Are you aware of the measures prescribed by the government for the control of COVID-19? Are they understandable? To what extent are you able to implement them?
  - At the level of your health district
- (follow-up question): How are the response activities going at the health district level? How are they coordinated? Is there any overlap? Are there important aspects that are not considered in the response at the district level? Are all the actors involved (including yourself and your facility)? Are all actors at the same level of information?
  - At the level of your main health facility
  - At your personal level, regarding your medical practice
- Are there any other challenges in the COVID-19 response that you would like to share with us?
- If the health district were to support you (and your facility) to better respond to COVID-19, what might that support be?
- What other support(s) would you need?
- What would you recommend for improving the COVID-19’s response in Benin?

## Part 2: Assessment of the impact of COVID-19 on the primary care physicians' work and the utilisation of primary care services

- - What changes did you notice in the utilisation of the services at your health facility since the advent of COVID-19 (changes in the utilisation rates, some services more used or less used, etc.)?
  - Which services have been most impacted by the pandemics, and how?
  - What changes have you observed in the health services organisation since the advent of COVID-19 (modification of opening hours, modification of consultation methods, absenteeism of health workers, more frequent stock-outs, etc.)?
  - What difficulties do you encounter in continuing to provide essential healthcare services to the population in the time of COVID-19’s?
  - How has COVID-19 changed your personal and professional habits?
  - What is the epidemic's impact on your morale and on your ability to continue working?
  - How is the community you are taking care of experiencing the pandemic and the changes it has brought about? Do you think that this pandemic contributed to changing the population's behaviour regarding healthcare? Please explain your answer.

## Part 3: Innovations in the health services organisation and lessons learnt

- - Which measures did you adopt to continue to provide essential services to your patients despite COVID-19?
  - Do you know of any other innovative measures implemented by other colleagues or other health facilities?
  - What would you recommend for avoiding or mitigating the impact of COVID-19 and its response on the management of other health problems?
    - (follow-up question) What can be done to improve the utilisation of health services in the context of COVID-19?
    - (follow-up question) What can be done to help healthcare providers (especially primary care physicians) continue to provide care to patients?
  - What are the main things you have learnt so far from the COVID-19 pandemic?
    - Do you think that COVID-19 will have medium-term or long-term consequences on the health system and your practice in particular?
    - Please share some lessons you have learnt.
