# Supplementary material for: Lessons Learnt From the Experiences of Primary Care Physicians Facing COVID-19 in Benin: A Mixed-Methods Study
Source: Front Health Serv. 2022 Mar 29;2:843058. doi: 10.3389/frhs.2022.843058 (PMC10012796; doi:10.3389/frhs.2022.843058)
Supplement: Supplementary file 3 [file Table_3.DOCX]

Supplementary Material 3

Supplementary table 1: Percentage of PCPs reporting the control measures implemented in their facilities, by the PCPs' categories

| Prevention and control measures | **PCPs’ category** | | | | | **Total**  **n (%)** |
| --- | --- | --- | --- | --- | --- | --- |
|  | **Public GPs**  **n (%)** | **Private GPs**  **n (%)** | **MGCs**  **n (%)** | **Specialists**  **n(%)** | **p-value^[[1]](#endnote-1)^** |  |
| Strengthening the cleaning of the facilities (N=87) | 6 (100.0) | 54 (100.0) | 7 (100.0) | 20 (100.0) | - | 87 (100,0) |
| Strengthening measures to prevent infections during the patient’s care (N=86) | 6 (100.0) | 53 (100.0) | 7 (100.0) | 20 (100.0) | - | 86 (100,0) |
| Installation of a hand washing facility at the entrance to FoSa (N=86) | 6 (100.0) | 52 (98.1) | 7 (100.0) | 20 (100.0) | 1.000 | 85 (98,8) |
| Availability of handwashing supplies (water, soap, hydroalcoholic gel) for healthcare staff (N=87) | 6 (100.0) | 53 (98.1) | 7 (100.0) | 19 (95.0) | 0.617 | 85 (97,7) |
| Providing COVID-19 information to patients (N=88) | 6 (100) | 50 (90.9) | 7 (100.0) | 19 (95.0) | 1.000 | 82 (93,2) |
| Providing COVID-19 information to healthcare staff (N=88) | 6 (100.0) | 50 (90.1) | 7 (100.0) | 19 (95.0) | 1.000 | 82 (93,2) |
| Availability of handwashing supplies (water, soap, hydroalcoholic gel) for users (N=87) | 5 (83.3) | 52 (96.3) | 6 (85.7) | 17 (85.0) | 0.131 | 80 (92,0) |
| Requiring the use of surgical mask for moderate risk staff (N=87) | 7 (100.0) | 46 (85.2) | 6 (85.7) | 18 (90.0) | 0.899 | 76 (87,4) |
| Sufficient provision of gloves (N=87) | 3 (50.0) | 48 (88.9) | 6 (85.7) | 18 (80.0) | 0.101 | 73 (83,9) |
| Requiring the use of gloves for the staff involved in triage or patient care (N=87) | 6 (100.0) | 47 (87.0) | 5 (71.4) | 14(70.0) | 0.175 | 72 (82,8) |
| Requiring the use of protective face masks or respirators for staff involved in triage or patient care (N=86) | 3 (50.0) | 38 (70.4) | 3 (50.0) | 12 (60.0) | 0.505 | 56 (65,1) |
| Sufficient provision of surgical masks for moderate risk staff (N=87) | 4 (66.7) | 34 (63.0) | 5 (71.4) | 10 (50.0) | 0.737 | 53 (60,9) |
| Availability of a protocol for dealing with a suspected COVID-19 case (N=87) | 5 (80.3) | 31 (57.4) | 6 (85.7) | 11 (55.0) | 0.352 | 53 (60,9) |
| Availability of a protocol for triage (N=88) | 4 (66.7) | 32 (58.2) | 4 (57.1) | 10 (50.0) | 0.926 | 50 (56,8) |
| Setting up a triage point (N=88) | 3 (50.0) | 32 (58.2) | 4 (57.1) | 7 (35.0) | 0.377 | 46 (52,3) |
| COVID-19 training for the staff (N=85) | 3 (50.0) | 24 (45.3) | 4 (57.1) | 10 (52.6) | 0.911 | 41 (48,2) |
| Reservation of a specific place in the facility for the consultation or isolation of suspected cases (N=86) | 4 (66.7) | 21 (39.6) | 1 (14.3) | 10 (50.0) | 0.234 | 36 (41,9) |
| Sufficient provision of face masks or respirators for staff involved in triage or patient care (N=87) | 1 (16.7) | 21 (38.9) | 2 (28.6) | 0.725 | 0.725 | 30 (34,5) |

1. Fisher’s exact test [↑](#endnote-ref-1)
