# Supplementary material for: Lessons Learnt From the Experiences of Primary Care Physicians Facing COVID-19 in Benin: A Mixed-Methods Study
Source: Front Health Serv. 2022 Mar 29;2:843058. doi: 10.3389/frhs.2022.843058 (PMC10012796; doi:10.3389/frhs.2022.843058)
Supplement: Supplementary file 4 [file Table_4.DOCX]

Supplementary Material 4

Supplementary table 4 : Percentage of measures reported by the institutional status and the localization within or outside the cordon sanitaire

|  | **Institutional status** | | | **Cordon sanitaire** | | | **Total** |
| --- | --- | --- | --- | --- | --- | --- | --- |
| Percentage of control measures reported by PCPs (N=81) | **Public** | **Private** | **p-value^[[1]](#endnote-1)^** | Within cordon | Outside cordon | **p-value^[[2]](#endnote-2)^** |  |
| Mean (SD) | 74.5 (19.0) | 74.9 (14.1) | - | 73.0 (15.0) | 78.4 (14.0) |  | 74.8 (14.8) |
| Median (IQR) | 77.8 (27.8) | 77.8 (16.7) | 0.7786 | 77.8 (16.7) | 80.6 (19.4) | 0.1368 | 77.8 (16.7) |

1. Wilcoxon rank sum test [↑](#endnote-ref-1)
2. Wilcoxon rank sum test [↑](#endnote-ref-2)
