# Supplementary material for: Lessons Learnt From the Experiences of Primary Care Physicians Facing COVID-19 in Benin: A Mixed-Methods Study
Source: Front Health Serv. 2022 Mar 29;2:843058. doi: 10.3389/frhs.2022.843058 (PMC10012796; doi:10.3389/frhs.2022.843058)
Supplement: Supplementary file 5 [file Table_5.DOCX]

Supplementary Material 5

Supplementary table 5: Percentage of measures reported by PCPs, by their practices categories

|  | **PCPs’ category** | | | | | **Total** |
| --- | --- | --- | --- | --- | --- | --- |
| **Percentage of control measures reported by PCPs (N=81)** | **Public GPs** | **Private GPs** | **MGCs** | **Specialists** | **p-value^[[1]](#endnote-1)^** |  |
| Mean (SD) | 76.8 (16.3) | 75.8 (13.9) | 75.0 (12.5) | 71.6 (17.7) | - | 74.8 (14.8) |
| Median (IQR) | 77.8 (11.1) | 77.8 (16.7) | 77.8 (16.7) | 77.8 (16.7) | 0.8879 | 77.8 (16.7) |

1. Kruskal-Wallis rank test [↑](#endnote-ref-1)
